# Notes on the Surgery of the War in the Crimea; with Remarks on the Treatment of Gunshot Wounds

**Published:** 1860-01

**Authors:** 


ART. II.—*Notes on the Surgery of the War in the Crimea; with Remarks on the Treatment of Gunshot Wounds.* By GEORGE H. B. MACLEOD, M.D., F.R.C.S., Formerly Surgeon to the Civil Hospital at Smyrna, and to the General Hospital in Camp before Sebastopol; Lecturer on Military Surgery in Anderson's University, Glasgow, etc. 12mo., pp. 439. London: John Churchill, 1858.

MILITARY surgery occupies, at the present day, a deservedly high rank in the estimation both of the profession and of the public. The war in the Crimea, the mutiny in India, and the recent convulsions in Italy, all attended with so much waste of blood and life, have invested it with a new interest. Its praises have been sung by Homer, and, in all ages of the world, governments have extended to it a fostering hand. As a distinct branch, however, of the healing art, it dates back no further than the early part of the sixteenth century, when it was inaugurated by Ambrose Paré, by the publication of his treatise on Gunshot Wounds, the fruits of his observations in the French army in Italy. This man, who was surgeon to four successive kings, was an eye-witness of the numerous French campaigns, from 1536 down to the battle of Moncontour, in 1569, a period of thirty-three years. His popularity, both as a civil and military surgeon, was, up to that time, without a parallel. The soldiers absolutely worshiped him; and the success of more than one siege was due almost exclusively to the wonderful influence of his presence. At the siege of Metz, the beleaguered garrison, exhausted by fatigue, disease, and famine, would have fallen an easy prey to the Spanish army, under Charles V., if Paré, suddenly appearing in their midst, had not reanimated their drooping spirits, and made them resolve to hold out at all hazards. Everywhere, as the news of his arrival was announced, the cry was, "Paré, our friend, is among us; we cannot die even if we are wounded." The treatise on Gunshot Wounds was published toward the middle of the sixteenth century, and, after having passed through various editions, was ultimately incorporated in Paré's surgical writings, published nearly a quarter of a century later.

In England, the earliest work on military surgery was that of Thomas Gale, entitled a "Treatise on Gunshot Wounds," designed chiefly to confute the errors of some of his contemporaries, respecting the supposed poisonous nature of these lesions. Gale was born in 1507, and after having served in the army of King Henry VIII., at Montrieul, and also in that of King Philip, at St. Quintin, finally settled at London, where he acquired great distinction in his profession. In 1639 appeared the work of J. Woodall, the Surgeon's Mate; or, Military and Domestic Surgery. He was surgeon under Queen Elizabeth, by whom he was sent to France, along with the troops that were dispatched to the assistance of Henry IV. and Lord Willoughby. In 1676, Richard Wiseman, serjeant-surgeon to King Charles II., published his famous "Chirurgical Treatises," one of which was expressly devoted to the consideration of gunshot wounds. Two years after this a treatise on gunshot wounds was published at London, by John Brown, also surgeon to Charles. He was a man of eminence, and served with much credit in the Dutch war of 1665. The next English work on military surgery appeared in 1744, from the pen of John Ranby, serjeant-surgeon to George II., under the title of "The Method of Treating Gunshot Wounds." After Ranby came the imperishable work of John Hunter, familiar to every reader of English surgical literature. The part relating to gunshot wounds was founded upon his observations made while serving as staff-serjeant at Belleisle and in Portugal, and is one of the most precious legacies of the last century, near the close of which it appeared.

The present century has been quite prolific in works on military surgery, as is shown by the valuable publications of Larrey, Hennen, Hecker, Augustin, Guthrie, Thomson, Hutchinson, Ballingall, Baudens, and others, which have contributed so much to the elevation of this department of the healing art. Some of these works have been reissued in this country, and have acquired a wide celebrity.

We must not forget, in this rapid enumeration of works on military surgery, the "Manuel de Chirurgien d'Armée" of Baron Percy, published at the commencement of the revolutionary war in France. It is a model of what such a treatise ought to be.

The only work on this department of science yet furnished in this country, is that of the late Dr. James Mann, published at Dedham, Massachusetts, in 1816. It is entitled "Medical Sketches of the Campaigns of 1812, '13, and '14," and forms a closely printed volume of upwards of three hundred octavo pages. Of this work it is our intention, ere long, to give a more particular notice.

Although the work of Mr. Macleod has been before the profession for some time, we do not deem it too late to introduce it to the notice of our readers; persuaded that a good book, like good wine, is never any the worse for the keeping. Moreover, the work needs no apology, either on account of the subjects of which it treats, or the manner in which these subjects are severally handled. It is a book to all intents and purposes; a great book within a small compass.

Passing over the first four chapters of the "Notes," which treat, respectively, of the history and physical characters of the Crimea, the construction and economy of camps, the campaign in Bulgaria, and its effects on the health of the troops, the distinction between military and civil surgery, and various other matters, as of no special interest to the American practitioner, we shall busy ourselves for a short time with the peculiarities of gunshot wounds and their general treatment, the topics discussed in chapter fifth.

The *sensation* and shock experienced in gunshot injuries are variable. The sensation accompanying a wound in a fleshy part is usually described by the soldier as resembling the effect of a smart blow from a supple cane. Occasionally, however, it is more like the impression produced by the passage of a red-hot wire. The pain is always greater when a bone is broken or splintered than when there is simply a wound of the soft structures, and more severe still when a large joint or visceral cavity is laid open.

The *shock* is not always, by any means, in proportion to the severity of the injury, depending, doubtless, much on the idiosyncrasy of the patient, the presence or absence of fear, and other causes, which it is not always easy to appreciate. Sometimes the slightest injury occasions the most profound depression both of mind and body, the patient looking ghastly pale, and quivering in every muscle, despite the most powerful efforts to control himself, and that, too, when he was not afraid to walk up to the cannon's mouth. At other times a man will have a limb carried off, or be injured in some vital organ, and yet hardly experience any shock at all; nay, perhaps, be hardly conscious that he has been seriously hurt. The author refers to the case of a soldier who had both legs shot away, and who declared that he knew nothing whatever of the accident until he attempted to rise.

"The destruction inflicted by a ball depends on the distance at which it is fired, the direction of its flight, its shape and velocity, as well as on the nature of the part struck. If fragments of metal are fired, as sometimes happened during the sieges of the Peninsula, as well as in the civil emeutes of Paris, and of which we had some experience in the Crimea also, a very lacerated, irregular, and dangerous wound may be caused. A ball passing at great speed over the surface of a limb may occasion a wound similar to that made by a knife. But this action of a ball is rare.

"The great velocity, peculiar shape, and motion of the conical ball give to its wounds a character considerably different from those which is present in wounds caused by a round musket-ball. If fired at short range, and if it strike a fleshy part, the conical ball produces, I think, less laceration of the soft parts than the old ball; but if the range be great, and the part stuck bony, with little covering of flesh, as in the case of the hand or foot, then the tearing, especially at the place of exit, is greatly more marked."

Mr. Macleod has not found it so easy, as he had been led to suppose from the accounts of authors, to distinguish between the *opening* of entrance and that of exit. His observations go to show that, while in many cases the former is more regular and less discolored than the latter, it rarely happens that the lips of one wound are inverted and those of the other everted. Much, in this respect, would seem to depend upon the speed of the ball, the distance at which it is fired, and the amount of resistance experienced in its transit through the body. If the velocity be great, and no bone be struck, the difference between the two apertures will be very trifling, both as it respects size and discoloration; under opposite circumstances, the wound of exit will always be considerably larger than the wound of entrance, especially when the ball is conical. When, on the contrary, the ball is fired close to the surface, and does not meet with any hard or resisting obstacle, the difference of size will be very small, and may even be in favor of the opening of entrance.

It is often a matter of great importance to determine, when two openings exist in a limb, whether they have been made by one ball, which has passed out, or by two balls, which are retained. The question is of grave importance both in a practical and in a medico-legal point of view, but its solution is, unfortunately, not always possible. Sometimes the openings of entrance and exit are materially modified by the introduction but non-escape of a foreign body, as a piece of breastplate, belt, or buckle, along with the ball, which alone passes out, or by the flattening of a ball against a bone, or its division by a bone into several fragments, each of which may afterwards produce a separate orifice. Generally speaking, the missile, at the place of entrance, carries away a piece of skin, and rends the skin where it escapes, the former being often found in the wound.

The *contusion* which the ball produces in traversing muscles, always causes the wound to suppurate and granulate; there are, of course, exceptions to this rule, as, indeed, we know from our own limited experience, but they are "few and far between." Muscles, badly injured by a ball, are very apt to become contracted during cure, unless special pains be taken to counteract this effect. Large shot and other foreign bodies sometimes lodge among the muscles, where they are occasionally retained for a long time without their presence being at all suspected. A tendon will often deflect a round but rarely a conical ball.

But the most disastrous effects of balls are upon *bones*, which are often, if not generally, fractured and splintered in the most frightful manner, each fragment becoming, perhaps, the source of additional injury. This is more especially true of the conical missile, which, unlike the round, is rarely turned out of its course, but passes on in a straight line, boring through soft part and bone as if they were so many planks. The consequence is that most of such cases either terminate fatally on the spot from hemorrhage or shock, or that they demand amputation. Indeed, the horrible ravages committed by the conical ball, as compared with the insignificant injury of the round, form one of the characteristic features of modern warfare. The conical ball is never split upon encountering a sharp, angular bone, as so often happened with the round ball prior to the general use of the former.

The *eccentric course* often pursued by balls in their passage round and through the body has been a matter of observation with all military surgeons. The subject, however, although of practical interest, need not detain us here

Mr. Macleod, with Begin and other eminent military surgeons, strenuously insists upon the importance of promptly extracting all balls and other foreign substances, if it be possible to get at them without inflicting too much injury, very justly alleging that their presence can only tend to keep up inflammation and promote discharge, if it do not produce other and more serious mischief. "In leaving," says he, "the ball unextracted, we never know what evils may follow. The keeping open of the wound exposes the patient, in the first place, to all the dangers of a life in hospital, and the very elimination of the foreign body by suppuration, if it takes place at all, necessitates a vast amount of annoyances."

"It is well to remember, also, in searching for balls, that they may have dropped out by the same aperture by which they entered, before we come to examine the case. Stromeyer has put us upon our guard against very curious errors, which he says he has seen made in cutting on the head of the fibula, and on a metatarsal bone for balls.

"Sir Charles Bell has shown how the nerves may indicate to us the position of the ball. In one case he found it by pressing on the radial nerve, and so discovering that the ball lay behind it. 'So when a ball has taken its course through the pelvis or across the shoulder, the defect of feeling in the extremity, being studied anatomically, will inform you of its course—that it has cut, or is pressing on a certain trunk of nerve.'"

The *fever* which supervenes upon gunshot injuries often puts on the character of an endemic or epidemic; but in war its tendency generally is to assume a low typhoid type. Hence nutritious food and drink, if not also tonics and stimulants, are often necessary at the very commencement of the treatment. When the system is exhausted by profuse discharge, cod-liver oil, with change of air, is eminently beneficial. Strict attention should be paid to the position of the wounded parts; cold is usually most grateful in the early stages of the treatment, but warm applications are always preferable when there is much inflammation, with a tendency to suppuration, or during the existence of abscesses. Poultices were rarely used in the Crimea, and in all cases the greatest attention was paid to the lightness of the dressings.

"The state of the weather has got much to do with the rapid cure of gunshot, as of all other wounds. From a perusal of the medical records of regiments serving in the colonies, it would, however, appear that hot weather, as in India, is, on the whole, more favorable than a cold climate."

Chloroform and hemorrhage constitute the topics of chapter sixth. A few words under each of these heads must suffice for our purpose. Speaking of some of the objections urged by some of his confrères against the use of *chloroform*, the author holds the following language:—

"For my own part, I never had reason, for one moment, to doubt the unfailing good and universal applicability of chloroform in gunshot injuries, *if properly administered.* I most conscientiously believe that its use in our army directly saved very many lives; that many operations necessary for this end were performed by its assistance, which could not otherwise have been attempted; that these operations were more successfully, because more carefully executed; that life was often saved even by the avoidance of pain; the *morale* of the wounded better sustained, and the courage and comfort of the surgeon increased."

The British employed chloroform almost universally; the French also used it very extensively; and Baudens declares that they did not meet with one fatal accident from it, although it was given, during the Eastern campaign, thirty thousand times or more. Surely there could be no stronger or more convincing proof of the safety of this remedy, which has, of late, been so much decried in certain quarters by men not always either too honest or too well informed.

*Hemorrhage* has always been the great dread of the military surgeon, and it may well be so when we recollect how often it is a source of death. When a considerable-sized artery is divided, it generally proves fatal on the spot, unless there has been great laceration and contusion of its tissues, when there may be almost an entire absence of bleeding. The author met with two cases, and he heard of some others, in which the legs were carried away by round shot, and no hemorrhage took place.

"The period at which consecutive bleeding is most apt to take place has been variously estimated. Guthrie sets it down as occurring from the eighth to the twentieth day, Dupuytren from the tenth to the twentieth, Henman from the fifth to the eleventh, and Roux from the sixth to the twentieth. In the cases I have myself observed, it has taken place between the fifth and twenty-fifth days, and, by a curious coincidence, it has appeared in the majority on the fifteenth after the receipt of the wound. In one case, a wound without fracture of the thigh, it was said to have taken place as late as the seventh week, and that when no gangrene or apparent ulceration was present."

Great danger frequently results from the mere grazing of an artery, the injured part often dropping off in the form of a slough, followed by fatal hemorrhage. A case is mentioned by the author where a patient bled to death at the end of the ninth day, during a fit of coughing, in consequence of the separation of a slough of the carotid, of the size of a pea, from injury sustained by the grazing of the tissues of the vessel, the sphacelus extending through its entire thickness.

Hemorrhage occurring soon after an accident was universally treated by enlarging the wound and tying both ends of the vessel. It was only when there was much swelling and matting of the parts that the main artery of a limb leading to the injured structures was ligated.

"There were many cases of hemorrhage from the hand succeeding gunshot wounds, which came under my notice during the war. Many of the injuries resulted from the accidental explosion of the patient's own gun, and, I suspect, in not a few cases from intention. Hemorrhage, in such instances, was at times very troublesome, especially when the bones of the hand were much fractured, as it was then difficult, if not impossible, to secure the vessel in the wound. The secondary bleeding usually appeared early in these cases, and, so far as my observation went, ligature of the brachial seems better practice, when local means fail, than putting a thread on the vessel of the forearm, as I saw done several times in the East. In recent cases we can often ligature the bleeding vessel, but in the sloughing stage, with a deep wound, and the bones much injured, it is impossible to secure it. To ligature the radial and ulnar separately, or conjointly, exposes the patient to operative dangers which bring no adequate return, as the probability of success is very small."

*Tetanus* was not a frequent consequence of wounds in the Crimea. Mr. Macleod could not learn the exact number of cases, but he was personally cognizant of only six that occurred in camp, and of seven which took place at Scutari. Mr. Alcock, in the Peninsular wars, ascertained that the usual proportion was as one to seventy-nine. In the Schleswig-Holstein campaigns, Stromeyer met with six cases in 2000 wounds. In the Hôtel-Dieu, at Paris, in July, 1830, there was but one instance of the disease among the 390 persons confined there on account of gunshot wounds. Larrey found tetanus sufficiently common in Egypt, but fails to give any definite ratio. Of 810 wounded men who came under the observation of Sir Gilbert Blane, in the West Indies, in 1782, thirty were seized with tetanus.

Nothing is more variable than the exciting cause of this disease; in some instances it follows the most insignificant scratch, while in others the most desperate injury is insufficient to provoke it. In the cases observed by our author, four supervened upon compound fractures of the lower extremity, four upon wounds of the thigh, leg, and face, the latter involving the destruction of the eye; one upon amputation at the shoulder, and one upon frost-bite of the toes, the primary lesion of the remainder not being known. In India tetanus was often provoked by unextracted balls, especially when lodged underneath strong fasciæ; and both in that country and on the continent of Europe the operation which seemed to give rise to it more frequently than any other was amputation at the shoulder-joint.

The effects of sudden vicissitudes of temperature in inducing the disease have long been familiar to surgeons. They are most striking in tropical regions, and when the change is from hot to cold, and from dry to wet. Larrey had repeated opportunities of observing the development of the disease under such circumstances, both in Egypt and Germany. After the battle of Bautzen, the exposure of the wounded to the cold night air produced over a hundred cases of tetanus, and a large number suffered from a similar cause after the battle of Dresden. Like effects were witnessed at Ferozepore and Chillianwallah. Baudens, in his treatise on gunshot wounds, states that the influence of cold and moisture in developing the disease, during the French campaigns in Africa, was most striking. Of forty slightly wounded men, placed in a gallery on the ground floor, during the prevalence of a northeasterly wind, fifteen were speedily attacked with tetanus. Similar effects have several times been noticed in this country. Thus, after the battle of Ticonderoga, in 1758, nine of the wounded who were exposed the whole night after the action, in open boats upon Lake George, died of locked-jaw; and during our war with Great Britain, most of those who suffered on board the Amazon, in the engagement before Charleston, were attacked with this disease a fortnight after, in consequence of a very sudden change of weather, the wind blowing cold and wet.

The extremes of heat and cold both favor the production of tetanus. In the East and West Indies, the slightest prick of the finger or toe is often sufficient to induce the disease, and the inhabitants of the Arctic region not unfrequently suffer in a similar manner. Dr. Kane, in his memorable expedition, lost two of his men from this affection, and he adds that all his dogs perished from a like cause.

The *mortality* from traumatic tetanus is notorious. Fortunate is he that recovers. "The cases occurring in our army," says the author, "have been, so far as I know, with one exception, universally fatal." Of seventeen cases reported by Alcock, only two were saved. Of thirty cases observed by Larrey, in Egypt, all died within a week of the appearance of the symptoms. The records of the army in India furnish the details of nineteen cases, of whom only one recovered. Baudens states that, in Africa, three out of fifteen escaped. Of the cases observed by Blane, in the West Indies, thirteen recovered and seventeen died. These remarkable differences in the mortality of the cases in the practice of this distinguished naval surgeon, is to be found, we suppose, in the greater mildness of the disease rather than in any particular skill in its management.

On reading, says Mr. Macleod, the many accounts that have been given of this disease, he is led to infer that the most reliable remedies are opium and chloroform. Baron Larrey placed most confidence in opium and camphor, with division of the principal nerve. During the

German campaigns, he is said to have treated several cases successfully after this method. Alcock cured two cases of seventeen reported by him; one apparently by opium alone, and the other by opium and large doses of carbonate of iron. Of the obstinate and hopeless nature of this disease, in its acute form, a correct idea may be obtained when it is stated that there is hardly an article of the materia medica that has not been faithfully tried, either alone or combinedly, without success, except in a few cases, which might possibly have gotten well without medicine of any kind Amputation has occasionally been followed by recovery, though generally it is worse than useless after the full establishment of tetanic spasms.

*Hospital gangrene* does not appear to have been either common or severe in the English army in the East, while the French suffered most dreadfully from it. During the first winter, it prevailed a good deal among the English soldiers, in a mild form, at Scutari, frequently attacking the openings both of entrance and exit of the ball in gunshot wounds. It did not appear to pass from bed to bed, but occurred rather as a sporadic affection, its favorite seat being the inferior extremities. It generally showed itself as an *ulcerous* disease, and its ravages were nearly always limited to the skin and subcutaneous cellular substance. Mr. Macleod never saw it attended by any marked gastric disturbance. It was noticed that those who had suffered from diarrhœa and scurvy were most obnoxious to its inroads. The most reliable local remedy was nitric acid, applied not only to the edges of the sore, but also to the healthy tissues for a short distance beyond. This had generally the effect of causing an effusion of lymph, which thus formed a barrier to the further extension of the morbid action. The local treatment was assisted by the exhibition of the tincture of the chloride of iron, in half-drachm doses, three times daily. The diet was always of the most generous nature, even when there was little constitutional depression.

The reason why the French suffered so much more frequently and violently from hospital gangrene than the English, is supposed to have been due to the fact that they always removed their wounded and operated men from the camp to Constantinople, that they were more or less injured in the transportation, and, lastly, that they were improperly crowded together in their ships and hospitals. The disease raged with extraordinary virulence and fatality in the hospitals on the Bosphorus. It also prevailed about the same time in the hospitals in the South of France; and it is asserted by Mons. Lallour, surgeon to the "Euphrate," that one vessel alone, in her voyage to the Bosphorus, was obliged, from this cause, to throw sixty bodies overboard in the short period of thirtysix hours. The malady was the true contagious gangrene, attacking not only open wounds, but cicatrices, and almost every stump.

The chief topical remedy of the French surgeons was the application of the actual cautery, as recommended by Pouteau and Delpech, the former of whom, as is well known, composed a most able and faithful account of this disease as it prevailed in the latter part of the last century in the Hôtel-Dieu, at Lyons, he himself having suffered from a violent attack of it. Our author states that it apparently arrested the morbid action. The application of the hot iron was assisted by the perchlorate of iron, charcoal, the tincture of iron, lemon-juice, and other adjuvants.

In the Crimea, during the heat of summer, in 1855, after the taking of the Quarries and the assault upon the Redan, the English surgeons lost a number of cases of amputation of the thigh, from moist gangrene, of a most rapid and fatal character. Most of them perished before the supervention of sphacelus, apparently overwhelmed by the violent poison which pervaded and prostrated the entire system.

"This form of the disease," says the author, "occurred in four cases under my own charge, in men who had had a limb utterly destroyed by round shot or grape. In all the knee-joints were crushed, the collapse was deep and prolonged, and the operation performed primarily in the middle third of the thigh. Three of the four were of very intemperate habits. All these cases took place about the same time, at midsummer, when many other similar cases appeared in camp. The wards, though full, were not overcrowded, and could, from their construction, be freely ventilated. The weather was sultry, and cholera was in the camp. The atmosphere was surcharged with electricity, and the dreaded sirocco prevailed. Wounds generally assumed an unhealthy aspect for days, when this pestilential wind blew. The cases of all those who died in my wards seemed to be doing perfectly well up to sixteen hours, at the furthest, before death. Three of them were seized on the eighth day after amputation, just as suppuration was being established. The fourth died on the fifth day. The seizure and consequent symptoms were identical in them all. In recording one case I relate all. During the night previous to death, the patient was restless, but did not complain of any particular uneasiness. At the morning visit, the expression seemed unaccountably anxious, and the pulse was slightly raised. The skin was moist, and the tongue clean. By this time the stump felt, as the patient expressed it, heavy like lead, and a burning, stinging pain had begun to shoot through it. On removing the dressings, the stump was found slightly swollen and hard, and the discharge had become thin, gleety, colored with blood, and having masses of matter like gruel occasionally mixed with it. A few hours afterwards, the limb would be greatly swollen, the skin tense and white, and marked along the surface by prominent blue veins. The cut edges of the stump looked like pork. Acute pain was felt. The constitution, by this time, had begun to sympathize. A cold sweat covered the body, the stomach was irritable, and the pulse weak and frequent. The respiration became short and hurried, giving evidence of the great oppression of which the patient so much complained. The heart's action gradually and surely got weaker, till, from fourteen to sixteen hours from the first bad symptom, death relieved his sufferings. All local and constitutional remedies which could be thought of were equally powerless,—nothing could relieve the system from the weight which seemed to crush it, or enable it to support the severe burden. Strong stimulants were the only remedies which appeared to retard the issue for a moment. Post-mortem examination, instituted shortly after death, showed the tissues of the limbs, and in many cases those of the internal organs also, to be filled with gas, and loaded with serous fluid. The vessels leading from the stump were healthy, and in only one case had there been any actual mortification previous to death. The intestines, in two of the four cases, were much diseased."

*Erysipelas* was not common in the Crimea, and generally readily yielded to ordinary treatment.

*Frost-bite* was very prevalent during the first winter which the English passed in the Crimea, and not a few perished from its effects. It was most common among those whose health had suffered from previous exhaustion rather than from the low degree of cold. The habit which the soldiers had of sleeping in their wet boots, which was at one time almost universal, from the fear that if they took them off they would not be able to draw them on again, contributed greatly to this result, the wet and cold combined diminishing the circulation and the vitality of the feet. The French suffered more from frost-bite than the English. In their hospitals, a limb, says Mr. Macleod, might occasionally be seen sphacelated half way to the knee. When the lesion was extensive, uncontrollable diarrhœa soon set in, and invariably destroyed the patient. According to the avowal of Mons. Scrive, not less than 2500 cases of frost-bite were admitted into the French ambulances, on the 21st of January, 1855, when the thermometer was at five degrees, and of these 800 died. Although the fact is not stated, it is probable that much of this extraordinary mortality was owing to the supervention of erysipelas and hospital gangrene. We infer this much from the circumstance that the surgeons were obliged to abstain from all operative interference.

The French found a solution of sulphate of iron the best topical remedy. Our author, on the contrary, derived most advantage from soothing applications. Any roughness or rude manipulation in dressing the injured parts was promptly resented by an increase of the local action, which not unfrequently terminated in gangrene, to which there was always a remarkable predisposition on any attempt to separate a toe or finger, however black and disorganized, and if attached only by a mere shred, was sure to cause great pain and constitutional irritation, often proceeding to an alarming extent.

Chapter seventh is devoted to an account of injuries of the *head*, and is one of the most interesting and instructive portions of the work. Lesions of this region of the body are extremely common among soldiers, and are, as Mr. Guthrie long ago remarked, "difficult of distinction, doubtful in character, treacherous in their course, and, for the most part, fatal in their results." This opinion of the celebrated English writer, long at the head of British military surgery, is amply sustained by the experience of past ages and by the recent revelations in India and the Crimea. Many who suffer in this way die either within a short period after the infliction of their injuries, if they do not perish on the spot, while those who escape their immediate effects are almost certain to fall a prey to secondary lesions; as inflammation of the brain and its meninges, disease of the biliary system, and metastatic abscesses, concerning which so much was written by some of the older surgeons. All men of experience are familiar with the danger of these injuries, and hence the importance of a wise and prudent prognosis.

Fracture of the skull by *contre-coup*, so common in civil practice, is seldom met with in military; for the reason, doubtless, that the injury is hardly ever inflicted upon the top or base of the cranium, as it is when a man falls upon his buttocks or is struck upon the vertex. The most frequent fracture is the punctured, but it is not the only one to which soldiers are subject, as large portions of the skull are often cut away by the sword or driven in by shells, grapes, and other missiles.

"The nature of the injury inflicted by a ball striking the skull will depend chiefly on the angle of incidence, and the velocity. The character of the ball, too, has more to do with the matter than is generally supposed." Thus, if the projectile strike the skull horizontally and forcibly, it will be likely to pass through its substance, and lodge in the brain, or make even two apertures; whereas if it strike obliquely and its force is partially spent, it will probably glance, merely grazing the bone and wounding the soft parts; or, it may be, make the circuit of the cranium. Now and then a ball has been known to be imbedded in the spongy substance of the skull, or to fracture the outer table without injuring the inner, and conversely; although the latter occurrence is one of great rarity. Mr. Macleod refers to a preparation, in the museum at Fort Pitt, which shows that the inner table may be considerably depressed without any corresponding lesion of the outer. The external wall of the frontal sinus has occasionally been knocked in without any injury to the internal. A conical ball does its work much more effectually than the old round ball; it is more apt to penetrate the bone, and generally causes a greater amount of comminution, scattering the fragments in every direction, and often producing instant death. Owing to the frightful havoc made by the conical ball, the punctured fracture is, the author thinks, less frequent now in military practice than formerly; nevertheless, he is disposed to speak with some degree of reserve upon this point, waiting for further opportunities for observation.

It is of great interest, in a juridical point of view, to know that a ball penetrating the skull from without generally, if not invariably, splinters the inner table much more than the outer. This is owing partly to the support that the outer table receives from the parts lying behind it, and partly to the diminished force of the ball in its outward passage through both plates. If the projectile perforate the skull at two points, the inner table at the opening of exit will retain its smoothness, while the outer will be torn up to an extent much larger than the ball.

Mr. Macleod refers to a case, which occurred at the Alma, where the skull was completely destroyed by a glancing shot, without any material implication of the scalp. A round shot ("en ricochet") struck the scale from an officer's shoulder, and merely grazed his head as it ascended. The result was instant death. The skull was so completely smashed that its fragments rattled under the scalp as if loose in a bag. The condition of the brain was, unfortunately, not ascertained.

One would naturally suppose that gunshot injuries of the skull would necessarily be attended, in almost every instance, with concussion of the brain; but the fact seems to be otherwise. It is only when the lesion is very great that evidence of concussion is apt to be present; under opposite circumstances it is either absent, or very slight and transient.

Sword-cuts sometimes slice away large parts of the skull, and yet such cases occasionally eventuate in the most brilliant recovery, the portion, if not wholly detached, and immediately replaced, firmly readhering. The author relates an instance, that of a Russian soldier, which was under his charge after the fall of Sebastopol, where the left parietal bone was cleft so as to be almost separated. No one, except his comrade, was permitted to touch him. His recovery was complete. In connection with this subject, Mr. Macleod mentions the remarkable rarity of hernia of the brain after sword, as compared with gunshot wounds:—

"The danger occasioned by gunshot wounds of the head will depend much on the part struck. At some places the ball is more apt to glance off than at others, while the strong processes of bone, the situation of blood-vessels, and the apparently greater necessity to life of some parts of the brain than others, introduce many elements into the calculation of the result. Notwithstanding all this, however, the curious eccentricities which characterize these injuries—the slight disturbance created by some, which, to all appearance and experience, are ten times more severe than others that prove fatal—upset our preconceived opinions; and, while they puzzle us to account for the difference, they prove the truth of Liston's aphorism, that 'no injury of the head is too slight to be despised, or too severe to be despaired of.'

"Generally speaking, it appears tolerably certain that wounds of the side of the head, especially anterior to the ear, are the most dangerous to life, and that a descending scale will give the following order: the fore part, the vertex, and the upper part of the occipital region, the last being decidedly the least dangerous. Remarkable exceptions to this graduating scale of danger do, however, occur.

"There are, at the same time, other circumstances besides the seat and nature of the injury which influence the result. The age of the patient is, perhaps, the most important of these. With children and young persons, the same gravity by no means attaches to the prognosis of head injuries, as to similar accidents occurring to the old. Mr. Guthrie has well observed, that in the accounts of wonderful escapes and successful operations on the head, the subjects have been, in general, below puberty. The temperament of the patient, his excitability, his social condition, as giving rise to more or less anxiety regarding the result of his case; the means there are of carrying out his treatment as to quiet, isolation, etc.; the place where he is treated, whether in the hospital of a populous city, where the results of such cases are usually so fatal, or in the country, where so much more can be accomplished,—all these are important items in forming an opinion regarding injuries of the cranium."

Speaking of operations on the head, there are, remarks Mr. Macleod, three classes of cases to which the trephine is still occasionally applied: "First, fracture with depression, before symptoms have appeared; second, fracture with depression, attended immediately with signs said to indicate compression; and third, fracture, with or without depression, followed at a later period by symptoms evidencing compression." In regard to the first of these classes of injuries, the experience of military surgery seems to be decidedly averse to operative interference. "There are, I believe," says the author, "very few surgeons of experience in the army now-a-days who approve of 'preventive trephining.'" A very large number of cases fell under his observation in the East, in which fractures of the skull, attended with extensive depression, rapidly recovered without any operative interference, simply by the use of evacuants and perfect tranquillity of mind and body. The language of Professor Macleod upon this subject is very pointed, and deserves to be reproduced here. "Every surgeon in the army," says he, "can recount many such cases. *If* any patients were lost from not having been operated on, I never saw any of them; but I do know of some patients who died, because they were subjected to operation." The accommodating powers of the brain in injuries of the skull have been a topic of remark from time immemorial; and the experience of almost all military surgeons goes to show that recoveries from these lesions are by no means unfrequent, even under apparently the most desperate circumstances, without operative aid, without any nursing, and even without any of the ordinary comforts of life, privation seemingly promoting the cure. Dr. Dease, of Dublin, in his able work on "Injuries of the Skull," long ago remarked that patients who totally neglected all treatment, and lived as they pleased, recovered quite as well as those who received every possible attention. Indeed, the chances of getting well, under such circumstances, are often much better, as the poor sufferers thereby escape the officious interference of the surgeon.

Great difficulty, as our author very justly remarks, usually exists in regard to the treatment when there is fracture with depression, immediately followed by symptoms of compression. When the depression is slight, and the symptoms not very urgent, he is of opinion that the best plan is non-operative interference, on the ground that by proper evacuants, repose, and light diet, the brain will gradually accommodate itself to the superincumbent pressure. If, on the other hand, the bone is driven deeply into the brain, and the patient is comatose, with stertorous breathing, a slow pulse, and a dilated pupil, an attempt at elevation should be made. It should not be forgotten, in trying to arrive at a proper conclusion in respect to the treatment of such cases, that the immediate cause of the compression may be effused blood; or, if some time have elapsed, exudative inflammation of the brain, or of this organ and its envelopes.

In the third class of cases, where the fracture, with or without depression, is followed at a late period by signs of compression, the hands of the surgeon are literally tied. All attempts at relief fail; inflammation has taken place, and the pus can seldom be evacuated; or, if evacuated, is not followed by any benefit.

In gunshot wounds of the head, perforating the skull, operative interference constitutes, according to Mr. Macleod, the least important item of the treatment. The examination should be made with the finger, and even that in the most cautious manner. The wound should not be enlarged, unless the object be to elevate depressed or remove loose bone. A piece of wet linen should be applied to the part, to exclude the air, and over this iced water, the head being well raised, and perfect quietude enjoined, with light diet, and an active purgative. The patient should be frequently visited, and if signs of cerebral inflammation arise, he should be promptly and copiously bled at the arm, and by leeches to the scalp.

In regard to the extraction of balls, when lodged in the brain, the almost universal practice in the army appears to be to get rid of them at once, if it be possible to seize them. The views of Quesney, Sir Charles Bell, and Sir George Ballingall are cited in favor of this procedure. Balls lodged in the brain occasionally become encysted; but such an occurrence is rare, and cannot, therefore, serve as a rule of practice. In nearly all cases their presence promptly determines fatal inflammation.

It is well known that portions of skull sometimes die after gunshot and other injuries. The practice among many able surgeons in the Crimea seems to have been to leave such cases to the efforts of nature, hoping for spontaneous expulsion. Our author adds that such treatment must often be very dangerous, an opinion in which we fully coincide with him. Nature can certainly do very little under such circumstances; dead bone—especially when loose, and fretting the dura mater—must necessarily act as a foreign body, which we have known, more than once, to cause epilepsy and other bad effects; and hence, we never hesitate to remove them as speedily as possible, since spontaneous expulsion is hardly to be expected in any case.

In a foot-note, Mr. Macleod has collected some interesting facts, illustrative of the mortality which attends the operation of trephining. Without entering into details, it may be stated, in general terms, that it is nearly always fatal.

Among the more common secondary effects of injuries of the head, spoken of by Mr. Macleod, is an intermittent headache, supervening at a variable period. A careful regulation of the diet and bowels, with vesication of the nape of the neck, and the exhibition of morphia, were found to be the best remedies. Jaundice was present in several cases; but the nervous irritation and weakness, so often noticed in civil practice, were rarely observed in the Crimea. The author did not meet with a single instance of hepatic abscess consequent upon head injuries.

A number of interesting cases are interspersed through this chapter, one of the most instructive and valuable in the book. We close our analysis of it with the following extract:—

"To multiply cases would be of little use. The teaching of all was to lead us to wait; to purge the patient thoroughly; to remove only such pieces of bone as could be got at with forceps, and which were quite detached and loose; to bleed, if need be, locally and even generally; to use cold applications, when there was a fear of inflammation; to enjoin perfect rest, not only to the body generally, but, if possible, to give repose to the special senses also, by isolating the patient, and thus removing the stimuli to their exercise; to enforce the lowest diet, and to continue all this treatment for a long period, even after all danger seemed past; and, finally, to treat any incidental complications on general principles."

Wounds of the *face* and *chest*, treated of in chapter eighth, need not detain us, inasmuch as the former present few points of interest, while we shall endeavor to dispose of the latter in connection with Mr. Patrick Frazer's monograph on that subject, in an early number of the Review The whole question has been ably discussed by both writers, and it will be our duty to exhibit their labors in as graphic and tangible a manner as possible.

The great source of annoyance and danger, in gunshot wounds of the face, is hemorrhage. It frequently appears early, and often stops spontaneously, though at times it is commanded with much difficulty. Paralysis, partial or complete, is not uncommon. The author alludes to the importance of preventing the swallowing of the secretions in wounds of the mouth and throat, asserting that they never fail to awaken constitutional irritation and typhoid fever. We have more than once witnessed similar effects in wounds of, and operations upon, the mouth, throat, and nose.

In fractures of the bones of the face from gunshot, an exception should be made to the general rule of removing fragments which are nearly detached, observation having demonstrated that the large supply of blood in this region will enable them to resume their connection with the other tissues, in a way that would be fatal to similarly placed portions in other parts.

The subject of gunshot wounds of the abdomen and bladder is discussed in chapter ninth.

Contusions of the walls of the *abdomen* by round shot are among the most dangerous injuries to which the body is exposed, often rupturing both the hollow and solid viscera, and rapidly causing death, without much apparent sign of so severe an accident. The most important symptoms of these contusions are vomiting and pain in the abdomen; and the great object of the treatment, in the event the patient survives their immediate effects, is the prevention of peritonitis, which often comes on in the most stealthy manner. Laceration of an internal organ is nearly always promptly fatal. Shell wounds of the walls of the abdomen are generally followed by extensive sloughing. Abscesses among the muscles of the abdomen are not uncommon after gunshot injuries.

Balls often traverse the walls of the abdomen for a considerable distance without entering its cavity, or entering without injuring any of the contained viscera. Many such examples are to be found in the writings of military surgeons. A very interesting one is briefly related by the author.

"The fatality of penetrating wounds of the belly will depend much on the point of their infliction. Balls entering the liver, kidneys, or spleen are well known to be usually mortal, although exceptional cases are not rare. Wounds of the great gut are also always recognized as much less formidable than those which implicate the small. Thomson saw only two cases of wounds of the small gut, after Waterloo, in the way of recovery; but Larrey reports several. Gunshot wounds of the stomach are also exceedingly fatal. Baudens records a remarkable case of recovery, although complicated with severe head injuries. The syncope which followed the severe hemorrhage in this case lasted for ten hours, and doubtless assisted, along with the empty state of the stomach at the moment of injury, in preventing a fatal issue."

Gunshot wounds of the *bladder* occasionally occurred during the war; the ball may penetrate the organ in any direction, and at the same time commit extensive havoc in the neighboring parts, both soft and osseous. Such lesions are generally fatal. Simple gunshot wounds, on the contrary, are sometimes recovered from, especially when they are treated by the retention of the catheter, thus allowing the urine to flow off as fast as it descends from the kidneys. Mr. Macleod does not refer, in connection with this subject, to the operation of laying open the wounded viscus through the perineum, as originally proposed by Dr. Walker, of Massachusetts. Such a procedure would be much more likely to prevent urinary infiltration than the catheter, however carefully retained, during the detachment of the sloughs, as well as before the contiguous structures have been glazed with lymph.

Balls, pieces of cloth, fragments of bone, and other foreign bodies, if retained in the bladder, generally serve as nuclei of calculi, and should, therefore, be as speedily extracted as possible, either through the perineum, or by means of the forceps or lithotriptor. Quite a number of cases, in which the operation of lithotomy was successfully performed for the purpose of effecting riddance of balls and other extraneous substances, have been reported by different writers, as Morand, Larrey, Baudens, Langenbeck, Guthrie, and Hutin. An interesting paper, detailing the particulars of thirteen cases, in ten of which balls were successfully removed, has been published by Mr. Dixon, in the thirty-third volume of the London Medico-Chirurgical Transactions. An abstract of this paper will be found in Professor Gross's Treatise on the Urinary Organs. In two cases, reported by Hutin, the ball was cut out; in one, at the end of nineteen, and in the other, after the expiration of thirty-two years.

*Compound fractures* of the extremities, and gunshot injuries of the hand and foot, form the subject of chapter tenth. Our limits will not permit us to do more than to quote a few brief passages from this portion of the work. The first refers to the dangerous character of compound fractures:—

"Gunshot fractures of the extremities of bone have always been considered dangerous, chiefly on account of the shock, the comminution of bone, and the fact that the wound leading to it is of such a character that it can heal only by suppuration, and cannot be so closed as to convert it into a simple fracture, which, it is well known, we can sometimes accomplish in such fractures as present themselves to us in civil practice. The cavity of the fracture is thus kept open to the air; the pus undergoes those changes which Bonnet has shown it does under such circumstances, and that severe and prolonged inflammation of the deep and irritable tissues which constitutes the chief danger in compound fractures, cannot be avoided. Now, all of these dangerous characteristics of compound fractures have been immensely increased by the conical ball. First of all, the shock it occasions is undoubtedly greater than that caused by the round ball, simply because the destruction it causes is much more severe. Secondly, the comminution of bone is enormously increased. The number of fragments which are quite detached are much more numerous, and the amount of sequestra, which are so far severed as to be ultimately thrown out before a cure can be looked for, is much greater. Thirdly, the bruising of the soft parts is more extensive, so that the suppuration is more prolonged, and the changes of purulent absorption so much the more multiplied."

The author dwells at considerable length upon the importance of early riddance of detached and partially detached fragments of bone, in cases of gunshot injury, alleging that their retention is always a source of suffering and severe suppuration, and, at times, even of death. That this is the correct practice, under such circumstances, is abundantly attested by the experience of all military surgeons. We quote the subjoined passages as expressive of Mr. Macleod's views upon this subject:—

"Finally, considering the question in all its bearings, it must appear pretty evident that the removal of fragments must tend immensely to simplify the wounds under consideration, and, therefore, that not only should all spiculæ which are entirely detached be removed as soon as possible, but that the same line of practice should be followed with regard to those which are so far detached as to retain but slight connections, and whose continued vitality must be doubtful; that this step should be accomplished by enlarging the exit wound; and that the practice is especially necessary in those cases where the femur is implicated, and a conical ball is the wounding cause.

"The tertiary fragments, or those extensively adherent, should, of course, never be interfered with. Parts of these fragments may subsequently exfoliate, but at what period this may occur it is impossible to say. They may not appear for months, or, it may be, for years. Mr. Curling has lately made the observation, that necrosed portions of bone in compound fractures are longer of getting loose when they are connected with the lower, than when they are attached to the upper part of the shaft."

Chapter eleventh treats of gunshot wounds and excision of the *joints*, and is replete with interest.

The gravity of lesions of this kind has been recognized by all practitioners, military and civil, from time immemorial. The principal circumstances influencing the prognosis are the size and complexity of the articulation, the extent of the injury, and the condition of the system. A gunshot wound of a ginglymoid joint is, in general, a more dangerous affair than a similar lesion of a ball and socket joint. The structures around the articulations often suffer severely, thus greatly adding to the risk of limb and life.

Speaking of gunshot wounds of the knee-joint, our author observes that the danger is not in the primary but in the secondary affections. "The long and wasting suppurations, the tedious and dangerous abscesses, and the purulent poisoning, are the principal sources of alarm." The matter of these abscesses forms almost invariably among the muscles of the thigh, and is extremely apt to burrow along the bone, stripping it of periosteum, and causing the most extensive havoc. During the progress of the disease, the knee-joint becomes enlarged, putting on all the appearances of white-swelling.

Military surgeons have long been agreed that when the articulating extremities of the knee-joint are fractured by a ball, the proper treatment is amputation, and the cases which occurred in the Crimea formed, it would seem, no exception to this opinion. In December, 1854, Mr. Macleod saw upwards of forty cases of this lesion in the French hospitals, and all, except one, in which the attempt was made to save the limb, proved fatal. The India Reports make mention of nine cases of a similar character, every one of which perished. Of sixty-five cases of gunshot wounds of different joints, related by Alcock, thirty-three recovered, but of these twenty-one lost the limb; and thirty-two died, eighteen without amputation. Guthrie never saw a patient recover from a gunshot wound of the knee-joint; and Esmach, who served in the Schleswig-Holstein wars, expressly declares that all lesions of this description demand immediate amputation of the thigh. The reviewer, a few years ago, met with a case in which a pistol ball penetrated the knee-joint and lodged apparently in the articular extremity of the femur, followed by an excellent recovery.

"Shell wounds of the knee are, as a whole, not so dangerous as bullet wounds. They frequently merely cut the soft parts open; or if they injure the bone, the larger aperture which they leave acts beneficially in permitting the free discharge of secretions. I have known many shell wounds in the neighborhood of the joint, and not a few in which the articulation was opened and even the bones injured, ultimately do well, so far as saving the limb goes; more or less anchylosis following."

Gunshot wounds of the ankle-joint generally did well, although they required long treatment. Several cases involving the shoulder-joint also made good recoveries. When a ball is impacted in the head of the humerus, Mr. Macleod advises its prompt removal, inasmuch as its retention would be sure to excite severe inflammation and caries, necessitating amputation or causing death.

The following table of excision of the joints is valuable. We copy it, with important alterations, for the sake of more easy reference:—

| Bones and Joints. | Cases. | Time. | Recovery. | Death. |
|---|---|---|---|---|
| Head of femur.......................... | 5 | Primary. | 1 | 4 |
| " " ........................ | 1 | Secondary. | ... | 1 |
| Knee-joint.............................. | 1 | Secondary. | ... | 1 |
| Os calcis, and part of astragalus... | 1 | ...... | 1 | .. |
| Head of humerus...................... | 8 | Primary. | 7 | 1 |
| " " ...................... | 5 | Secondary. | 5 | .. |
| Ditto, and part of scapula.. .. | 1 | Secondary. | ... | 1 |
| Elbow-joint............................. | 13 | Primary. | 10 | 3 |
| " " ............................ | 4 | Secondary. | ... | 4 |
| Ditto, partial........................ | 3 | ...... | 3 | .. |
| | 42 | | 27 | 15 |

It is proper to add that the cases of secondary resections of the elbow-joint perished from causes not connected with the operation. Larrey performed excision of the shoulder, in Egypt, ten times, with six recoveries. Baudens cured thirteen out of fourteen cases; Legouest four out of six; the Schleswig surgeons twelve out of nineteen. Percy, in 1795, reported nineteen cures after resection of the shoulder.

The above results are eminently encouraging, and reflect great credit upon the skill and judgment of the different surgeons under whose care they took place. Mr. Macleod states that partial resections, of which there were a good many cases, did not, on the whole, turn out nearly so well as complete ones. He says they were more tedious, more liable to fail, and less satisfactory when they succeeded, than when the whole joint was removed.

The hip was resected six times in the Crimea; first by Mr. Macleod himself, and afterwards by some of his colleagues. Of these cases, five were primary and one secondary, one of the former alone succeeding. Of twenty-three amputations at the hip-joint by the English and French surgeons, not one recovered. Besides, it was found that the resected cases lived longer, on an average, as well as in much greater comfort, than the amputated, thus throwing the preponderance decidedly in favor of the former operation.

Excision of the knee-joint was performed only once; it was rendered necessary by a gunshot wound, and terminated fatally on the twentieth day.

Resection of the bones in their continuity was not much practiced in the Crimea. The results were not very flattering. The operation, as practiced upon the femur, was invariably fatal.

The last chapter of Mr. Macleod's work treats of *amputations.* The results of the military surgery in the Crimea show that the success of these operations, when performed early, was very fair, while, when undertaken late, it was most unfortunate. This was the case in both armies. Chloroform is said to have contributed greatly to the success of primary amputations, which became more frequent during the progress of the war, experience having proved that many patients, whose limbs were attempted to be saved, were lost in consequence of the delay.

A singular fact has been noticed by military and naval surgeons in regard to the absence of "shock" immediately after the infliction of gunshot injuries. Mr. Hutchinson was one among the first to call attention to this remarkable phenomenon, which has been frequently observed by others. Mr. Macleod was struck with it in several instances in the Crimean war, even after so severe a lesion as the loss of a limb, and he lays much stress upon it, in reference to the favorable opportunity which such an event is calculated to afford for the performance of amputation.

> "It is during this interval, too, that we obtain the full good of the soldier's *moral* advantages over the civilian. 'Cut off the limb quickly,' says Wiseman, 'while the soldier is heated and in mettle'—and the observation is as old as Paré, that while excited by the combat, and yet within sound of the cannon, the soldier or sailor willingly parts with a limb which a few hours of reflection would make him desire to run the risk of preserving, and upon which he fixes all his attention, so as to magnify greatly the dangers of the subsequent operation. Moreover, the removal of the man, before operation, to any distance from the scene of his accident, lessens somewhat his chances of recovery; as, besides the danger that the irritation and pain of such transport, however carefully it may be conducted, will occasion, the constitutional depression we dread, the mere loss of blood which, although going on in very small quantities, is yet flowing in drops, when a drop may extinguish life, are serious objections to the shortest delay."

One of the difficulties in the way of immediate amputation is the want of recognition of the cases demanding the operation, and the uncertainty that none of the internal organs have sustained any fatal injury, a circumstance which would, of course, contra-indicate the propriety of surgical interference.

Mr. Macleod expresses himself as a decided advocate of the circular operation, and it is not a little singular that most of the army surgeons of this and other countries regard it as preferable to the flap. It is well known that Sir Charles Bell termed the circular method "the perfection of the operation of amputation."

Mr. Macleod has furnished some very interesting statistics of amputations, which we shall endeavor to transcribe here for the benefit of the reader. In doing this, it is proper to state that the figures refer solely to the period between the 1st of April, 1855, and the close of the war, excluding thus all the unfavorable part of the campaign, as well as most of the operations performed during the actual contest. During this period 732 primary amputations took place, with a mortality of 201. Of these, 654 were primary, with 165 deaths, or 26·22 per cent.; and 78 secondary, with 36 deaths, or in the ratio of 46·1. The mortality of the greater amputations—as those of the shoulder, arm, and forearm, and the hip, thigh, knee, and leg—was 39·8 per cent. for the primary operations, and 60 per cent. for the secondary.

The increase of mortality from amputations as we approach the trunk, has long been familiar to surgeons, and the results in the Crimea have not changed our previous knowledge. Thus the ratio of mortality of amputations of the fingers was 0·5; of the forearm and wrist, 1·8; of the arm, 22·9; of the shoulder, 27·2; of the tarsus, 14·2; of the ankle-joint, 22·2; of the leg, 30·3; of the knee-joint, 50·0; and of the thigh, in its lower third, 50·0, at its middle, 55·3, at the upper part, 86·8, and at the hip, 100·0. The limb was removed at the hip-joint in 10 cases, all of which rapidly proved fatal. The French had 13 cases, primary and secondary, with no better luck.

Mons. Legouest has published a table of most of the recorded cases of amputation at the *hip-joint*, for gunshot wounds. Of these 30 were primary, and all ended fatally; of 11 intermediate, or early secondary, 3 recovered; and of 3 remote, 1 recovered. "Thus," says our author, "if we sum up the whole, we have 4 recoveries in 44 cases, or a mortality of 90·9 per cent." Some of the primary cases died on the table; and all the rest, except two, before the tenth day. In the Schleswig-Holstein war, amputation at the hip-joint was performed seven times, with one cure. Mr. Sands Cox, recording the experience of civil and military hospitals up to 1846, gives 84 cases, most of them for injury, with 26 recoveries. Dr. Stephen Smith, of New York, has published tables of 98 cases, showing a ratio of mortality of 1 in $2\frac{3}{5}$. In 62 of these cases, the operation was performed in 30 for injury, with a mortality of 60 per cent.

Amputation in the upper third of the *thigh* was performed 39 times, with a fatal result in 34. Of these cases only one was secondary, and that perished. Amputation of the middle third of the limb was performed in 65 cases, of which 38 died. Of these cases 56 were primary, with 31 deaths, giving thus a mortality of 53·3 per cent.; 9 cases were operated upon at a later period, and of these 7 died, or 77·7 per cent.

Amputation of the lower third of the thigh was performed 60 times, 46 being primary, with a mortality of 50 per cent., and 14 secondary, with a mortality of 71·4 per cent.

Amputation at the *knee* was performed primarily in 6 cases, of which 3 were fatal, and once secondarily, with a fatal result. Chelius refers to 37 cases of amputation of the knee, collected by Jæger, of which 22 were favorable; and of 18 cases recorded by Dr. Markoe, of New York, as having occurred in the practice of American surgeons, 13 got well. These cases, added together, afford an aggregate of 61, with a mortality of 21, or 34·4 per cent.

The *leg* was amputated 101 times, with 36 deaths, or a mortality of 35·6 per cent. Of these cases 89 were primary, with 28 deaths, and 12 secondary, with 8 deaths.

Amputation at the *ankle-joint* was performed in 12 cases, death following in 2. Of these cases 3 were secondary, and all favorable. Of Pirogoff's operation, Mr. Macleod thus expresses himself:—

"Pirogoff's modification of Syme's method was, I understand, several times tried at Scutari. I saw none of these cases, and am ignorant of the results. In England it appears to have been recently followed by good effects in six out of nine cases in which it was performed. Langenbeck is said to approve of its results in a good many cases in which he has tried it; but the history of the three cases first reported by M. Pirogoff himself, and those more recently put on record by Michäelis, of Milan, and various German surgeons, does not hold out much encouragement to repeat the operation, not only from the long period necessary to a cure, but also from the unsatisfactory nature of the resulting member. It was reported in the East that this operation had been frequently performed by Pirogoff himself in Sebastopol, but that he had found the calcaneum act as a foreign body in the stump, and was hence disposed to abandon it."

The arm was removed at the *shoulder-joint* in 39 cases, with a fatal issue in 13, or 33·3 per cent., 33 being primary, with 9 deaths, and 6 secondary, with a fatal issue in 4. If we couple these cases with 21 that occurred during the previous period of the war, we shall have an aggregate of 60 cases, with 19 deaths, or a mortality of 31·6 per cent. The advantage of primary over secondary amputation of the shoulder has long been known to military surgeons. Thus, of 19 primary cases mentioned by Mr. Guthrie as having occurred between June and September, 1813, 18 recovered, while of 19 secondary cases 15 died. The experience of the late Dr. Thomson, in Belgium, is equally decisive.

Amputation of the upper *arm* was performed 102 times, with death in 25 cases, or a mortality of 24·5; 96 of the cases being primary. Of the 6 secondary cases one-half proved fatal.

The *forearm* was amputated, primarily, 52 times, and the hand at the wrist once, with only one death; while of 7 secondary operations in the same parts 2 died.

"The mode of managing stumps in the East was that usually followed at home for the promotion of adhesion by the first intention. The edges of the flaps were usually united by suture. The observation of this method in the Crimea did not certainly appear to be satisfactory. To wait, as Liston so strongly advocates, till all oozing has ceased from the cut surfaces, is unquestionably a most useful precaution, and one of great moment to their successful and early union. The irritation which the stitching of the edges occasions, the want of sufficient room for subsequent swelling, the confinement of pus which is thereby favored, all appear reasons against sutures. Stripes of wet lint, applied like adhesive plaster, always appeared preferable. I never saw one case among our most numerous amputations in which primary adhesion took place throughout the whole surface of the flaps. They united readily enough along their edges; but the result of this was, that a large bag of pus was formed within the end of the stump, which continued as a depot for absorption into the system, by steeping the end of the sawn bone and the vessels in its matter, and it burrowed far and wide in the intermuscular spaces and along the bone, and ended not unfrequently in causing considerable necrosis of the end of the divided shaft. Unquestionably it may be said that such collections should have been recognized and prevented; but yet it seems to me that when ample proof is afforded, as it was early in the East, that primary adhesion was the rare exception, and not the rule, and when the patients were so peculiarly liable to purulent absorption as they were with us, it would have been better practice not to have attempted primary union, but to have adopted such treatment as best favored the freest discharge of the matter so soon as it was formed. The method of dressing with compresses, recommended by Mr. Luke, was most useful in several cases in which I tried it, in preventing the accumulations referred to. The contrast afforded by the heavy dressings for stumps employed by the French and our water dressing was very marked, and may have contributed something to the result which obtained in the less prevalence of purulent absorption in our hospitals than with them. Bad as it was with us, it never became the terrible epidemic it was in the French hospitals. We had no means of trying the method of treating stumps in water, recommended by Langenbeck. The ease with which the purulent secretion can be got quit of by position in amputations of the arm and leg contributes, I have no doubt, not a little to the decreased mortality attending these operations, as compared to amputations of the thigh. The Russian surgeons, I am told, when operating by the circular method, which they generally adopt, split the posterior flap, and keep this part open in order to drain off the pus. Such a step would meet with little favor in this country, but it presents many advantages when purulent absorption is so common as it was in the East. M. Sédillot, of Strasburg, I believe proposes a similar modification for general use."

The chief source of the mortality after amputation in the East, was *purulent infection.* This was especially true of the secondary operations. Many beautiful examples presented themselves, on dissection, of veins leading from the stumps retaining their round, patulous condition, while their canals were filled with pus, and their coats sometimes reddened. "*It was not uncommon to trace the pus-filled vein from the thigh to the vena cava.*" The secondary deposits were, as usual, generally found in the lungs. In the Holstein war, Beck found the lungs thus affected in seven cases out of ten in which death was caused by pyemia. The French surgeons at Constantinople found, on the contrary, the pus seldom collected in depots, as they had been accustomed to see it in Africa, but disseminated through the viscera, muscles, and bones.

The visceral congestions which so often follow the larger amputations were more than commonly fatal in the Crimea, owing, as was supposed, to the presence of pre-existing disease in the lungs, kidneys, and intestines. Phthisis and acute dysentery were not unfrequently the immediate cause of death after amputations.

We have thus brought to a close our notice of this able, interesting, and instructive work, which we have no hesitation in pronouncing one of the most valuable contributions to military surgery ever made by any author. Our review, it will be perceived, is altogether of an analytical character, for we have been desirous rather that Mr. Macleod should speak than that we should speak for him. The work is written with great clearness and ability, and must serve as an enduring monument of the author's industry, discrimination, and power of observation. He now holds the appointment of Professor of Military Surgery in Anderson's University, Glasgow, and we may therefore confidently anticipate other works from a pen that has already achieved so much. We tender to Mr. Macleod our cordial acknowledgements for the "Notes" of which we have given so elaborate an account, and by the study of which we have ourselves so much profited.

We had intended, in connection with Mr. Macleod's work, to offer some remarks on Dr. Henderson's "Hints on the Examination of Recruits of the Army," a new edition of which was published a few years ago by Dr. Richard H. Coolidge; but as we have already transcended our limits, we are reluctantly compelled to postpone our notice, as also an examination of Dr. Trippler's "Manual of the Medical Officer of the Army of the United States," to another time.

